# Report of Interesting Operative Cases at Presbyterian Hospital

**Published:** 1903-08

**Authors:** E. C. Davis

**Affiliations:** Atlanta, Ga.; Gynecologist to Presbyterian Hospital and Tabernacle Infirmary


### REPORT OF INTERESTING OPERATIVE CASES AT PRESBYTERIAN HOSPITAL.*

BY E. C. DAVIS, M.D., ATLANTA,

Gynecologist to Presbyterian Hospital and Tabernacle Infirmary.

In casting about for a subject to present to the members of this staff it occurred to me that a brief report of a few of the more important cases coming under my care at the Presbyterian Hospital would prove more interesting than anything which I could offer. I make no effort to include all coming under my care, but have selected a few which represent in many instances a class of operations, and it would be uninteresting to report all of the class.

CASE 1.—Mrs. F., aged twenty-three, Georgia. Referred by Dr. W. K. Johnson. Mother died of tuberculosis. Menses first at fifteen, irregular, would miss two or three months at a time; last occurred two weeks prior to entering the hospital. Dysmenorrhea, pains in right and left inguinal region, shooting down

Paper read at meeting of Presbyterian Hospital Staff, June 30, 1903

thighs and in lumbar region. Intermenstrual pains constant. Walking or standing causes bearing down pain. No children. Leucorrhea constant, began at fifteen years of age, thick, white, worse just before menstruation. Examination revealed small ovarian cyst with endometritis. Uterus enlarged, prolapsed and retroverted. Patient had been treated for years with tampons, iodine, etc., without relief. Both tubes and ovaries were removed and uterus brought forward by shortening round ligaments, after a thorough curettage. Her recovery was uneventful, and while nearly two years have elapsed she is now attending her household duties, thoroughly relieved.

CASE 2.—Mrs. S. A. M., aged thirty-one, Georgia. Has always been nervous. When about seventeen years of age began to have marked hysterical manifestations, would faint and complain of being blind. When twenty-one years of age, about one year after marriage, was seized with chill pain in left side, together with several convulsions. Has never been free from pain in side since. Now complains of pain in back and left side. Began menstruating at seventeen years of age. Has always been regular. Dysmenorrhea, severe pain in right and left inguinal region over lumbar region, constant bearing down in character. One child nine years of age. Labor normal, getting up well. Examination revealed laceration of perineum and cervix uteri, enlarged, prolapsed, retroverted uterus, both ovaries prolapsed, resting low down in the pelvis, the left bound down by firm adhesions. Operations:—Perineorrhaphy, trachelorrhaphy, curettage, double salpingo-oophorectomy with shortening of round ligaments. Patient made a most excellent recovery and continues well.

CASE 3.—B. M., Alabama, aged twenty-two. Referred by Dr. Cromer. Complained of constant pain in back and left side. Menses first at seventeen, regular, lasting seven days, amount excessive; last occurred about a week previous. Dysmenorrhea, severe pain in right and left inguinal and lumbar regions, shooting down thighs. Pain constant. Worse during the flow. In bed three or four days. One child two years of age. Labor tedious, in bed thirty-two days, getting up ill. Urinates two or three times during the night and very frequently during the day. Examination showed two markedly enlarged ovaries low down in pelvis, uterus subinvoluted and prolapsed, laceration of cervix and perineum. Operation salpingo-oophorectomy, shortening round ligaments. The patient being quite weak the plastic operations were deferred. She left the hospital without having these attended to. She was greatly improved at first, but became very imprudent and developed some gastro-intestinal trouble from which she was suffering when I last heard of her.

CASE 4.—Miss M. S., Georgia, aged twenty. Referred by Dr. Garlington. Mother suffered from some uterine trouble, for which she underwent an operation. Nine months ago had la grippe, from effects of which she has never recovered. Has been in bed almost constantly since, suffering from pain in side, especially the left, very weak, emaciated and nervous. Menses first at thirteen, always irregular, from fourteen to sixty days, lasting from two to seven days. Dysmenorrhea, severe pain in both inguinal regions, shooting down thighs, constant for past eight months. Intermenstrual pain constant. Leucorrhea constant. Examination showed two small, prolapsed, cirrhotic ovaries, which were removed. The patient made a good recovery and walked without any discomfort.

CASE 5.—C. J., aged twenty, North Carolina. Mother died of phthisis. Has had pain in lower abdomen and back since birth of child fourteen months ago. Menses first at fifteen years of age, irregular, occurring two to six weeks. Dysmenorrhea, severe pains right and left inguinal and lumbar regions, constant, worse one week before flow begins. Intermenstrual pains constant. One child fourteen months old. Leucorrhea first at sixteen ; constant since. Examination, uterus prolapsed, retroverted and subinvoluted ; two small ovarian cysts. Operation, curettage, double salpingo-oophorectomy and shortening round ligaments. Recovery good. Patient is at work daily.

CASE 6.—Mrs. H., aged thirty-two, Georgia. Referred by Dr. Couch. Father living, mother died of phthisis. Has been suffering with pain in head, lumbar right and left inguinal region and lower part of abdomen. Menstruation first at thirteen, usually occurs every twenty-one days. Last occurred one month ago. Dysmenorrhea, severe pain right and left inguinal and lumbar regions, worse one week before flow begins. One child ten years old. Labor tedious. In bed three weeks, getting up ill. Leucorrhea first at eleven, worse after menstruation, extremely nervous and sleeps badly. Examination revealed a boggy mass behind the uterus and extending up on both sides, great tenderness. Temperature 98, pulse 80 to 96. Operation, celiotomy for ectopic gestation, tubal with rupture of tube. Both ovaries and tubes diseased. Double salpingo-oophorectomy and appendectomy. Patient made an excellent recovery and is now attending to her domestic duties.

CASE 7.—Mrs. W. N. J., Georgia. Five months previous to entering the hospital was delivered of still-born child. Profound sepsis supervened, and an enlargement was observed about one month after confinement, beginning on the right near iliac crest. This increased in size until it extended above the umbilicus. Patient became profoundly septic, having high temperature with chills and copious sweats. Her skin was dark and yellow. This fluctuating tumor had been incised by the attending physician at the highest point, viz. the umbilicus, and at least a quart of pus was reported to have been evacuated. This soon closed and has ruptured three times since. When brought to the hospital her condition was very unfavorable; she had a large fluctuating mass extending two inches above umbilicus, with a small sinus discharging nauseous, offensive pus through the umbilicus. She was thoroughly septic, her skin almost a mahogany hue. The abdominal muscles were board-like. Laparotomy was performed and an enormous abscess removed ; abdominal cavity was thoroughly irrigated with hydrogen dioxide and normal salt solution, abdomen was drained with gauze, and patient put to bed in a very weak and unpromising condition. She reacted promptly and made a most excellent recovery, except for small sinus in abdominal wall, which had not healed when I saw her last. She was then in good health, with a clear, rosy complexion. This was to me one of the most interesting of the cases coming under my care at the hospital.

CASE 8.—Miss T. Referred by Dr. Greene. When seen by me was suffering from pelvic peritonitis with pelvic abscess. Patient was removed to hospital and a colpotomy performed, evacuating fully a pint or more of pus. Recovery was good and more than a year has passed and patient is actively engaged as a stenographer in large office in the city.

CASE 9.—Mr. J. Was called about 4 a. m. and found him suffering from severe attack of appendicitis. Had him removed to hospital and operated on same day. Found a large, inflamed appendix, which was removed, McBurney's operation being performed. Patient left the hospital on fourteenth day and has had no subsequent trouble.

CASE 10.—Mr. V. Referred by Dr. Cromer. Patient was attending church service when seized by sudden pain in right side, which became so severe that he called to see Dr. Cromer, who diagnosed it as ruptured appendix and urged immediate operation, which was performed at the hospital. This was first attack as far as could be determined. The appendix was found gangrenous and ruptured and beginning peritonitis. The patient developed an ether pneumonia and was extremely ill for several days; made a good recovery and is now actively engaged in his usual duties. Several similar cases of appendicitis have been operated on, but the two above illustrate the principal types which have been encountered.

CASE 11.—L. Y., aged thirty-three. Referred by Dr. Cromer. Mother died from uterine hemorrhage, probably malignant disease of cervix. Patient suffered from pain in both inguinal regions and hypogastric, pain worse on left side. Menstruation usually regular, but for past three months has constant flow of blood. Drugs and douches exerted no benefit as to checking flow. Dysmenorrhea, severe pain inguinal both sides, shooting down thighs; had to go to bed one day before each menstruation. Intermenstrual pains constant. One child nine months of age. Labor tedious, instrumental, in bed three weeks. Examination revealed enlarged, soft uterus with a constant flow of blood. With mother's history and the suspicious appearance of the uterus, and both tubes and ovaries being diseased, a panhysterectomy was performed. Patient made a most excellent recovery and was actively engaged in her duties as cook when last heard from.

CASE 12.—Mrs. T. M. B., aged thirty-two. Referred by Dr.

Couch. Mother died at fifty years. Had an ascites from some cause. One brother died from phthisis. Menses began at fifteen years of age, irregular, amount excessive, lasting from three to seven days. Dysmenorrhea, severe constant pain in right and left inguinal regions, shooting down thighs, hypogastric lumbar, bearing down, worse two to three days before the menstrual flow begins. In bed from nine to ten days each month. Walking and standing causes bearing down sensation. Children five, oldest ten years, youngest one year; abortion thirteen months ago, in bed seven weeks; infection, getting up ill. Leucorrhea constant, bloody, worse before menstruation. Examination revealed both tubes enlarged and adherent by inflammatory bands to pelvis and viscera; uterus enlarged with chronic endometritis; both ovaries low down in pelvis and bound by adhesions. Panhysterectomy was performed, and as appendix was found diseased it too was removed. Patient made a good recovery and has since been attending to all her household work, cooking, etc. This patient had spent years in having local applications made and had not been improved a particle, but had grown steadily worse until she had become almost a physical wreck.

I will not prolong this report, as already it has outgrown my original intention, but hope this brief condensation will prove interesting. I have been assisted in these cases by Drs. Ellis, Moncrief, Fisher, Sutton and Barnett.

